# MST-G: Micro Suction Tape Gripper Climbing Robot with Active Detachment Capability

**DOI:** 10.3390/s24237790

**Published:** 2024-12-05

**Authors:** Jichun Xiao, Jiawei Nie, Lina Hao, Zhi Li

**Affiliations:** 1 State Key Laboratory of Synthetical Automation for Process Industries, Northeastern University, Shenyang 110819, China; 2 School of Mechanical Engineering and Automation, Northeastern University, Shenyang 110819, China

**Keywords:** climbing robot, micro suction tape, active detachment, adaptive grasping

## Abstract

Effective adaptive grasping capability is regarded as crucial for climbing robots. However, many dry adhesion legged climbing robots are primarily focused on mobility and load capacity to perform various climbing tasks, often overlooking their operational grasping abilities. Furthermore, flexible grippers designed for adaptive grasping are typically not capable of supporting autonomous climbing or perching motions; they must be rigidly integrated with legged climbing robots, which results in increased weight and reduced load capacity. To address this challenge, a novel dry adhesion climbing robot, MST-G, is proposed, featuring autonomous climbing, perching, and flexible adaptive grasping capabilities. During operation, MST-G is integrated with a legged climbing robot to perform tasks, but can autonomously climb when no task is present, thereby reducing load and ensuring stable motion. Additionally, a robust controller based on prescribed performance is introduced and tested on MST-G, which limits the joint tracking error to a prescribed safety limit, ensuring that motion trajectories can be executed safely and reliably.

## 1. Introduction

Climbing robots show great promise for applications in various fields, such as ship cleaning [1,2,3], bridge inspection [4,5,6,7,8], and power equipment repair [9,10,11,12]. They are capable of performing specific tasks that other types of robots cannot accomplish. For instance, Harvard’s HAMR-E [13] is designed for complex mechanical tasks such as inspecting and maintaining jet engines. The MARVEL [14] quadruped climbing robot from the Korea Advanced Institute of Science and Technology can perform inspection tasks on the surfaces of oil storage tanks. Legged climbing robots can be categorized based on their adhesion mechanisms into magnetic [14,15,16], electrostatic [17], negative pressure [18,19], and dry adhesive types [20,21,22]. Compared to other adhesion methods, dry adhesion allows robots to perch on surfaces for extended periods without energy consumption, offering a more significant advantage. To maximize the adhesive load-bearing capacity, non-directional dry adhesive materials like micro-suction tape (MST) are undoubtedly the preferred choice for dry adhesive climbing robots, which have higher adhesive strength and lower cost, and can adhere to surfaces at arbitrary angles. However, when detaching from the adhesive surface, a greater detachment force is generated, which hinders the smoothness of the detachment process.

Current developments in dry adhesion-based legged climbing robots have largely focused on enhancing locomotion and load-bearing capabilities. For example, the Geckobot [23] achieved vertical surface climbing with a maximum unloaded speed of 1 cm/s. Similarly, the Stickybot/RiSE [20,24] series of legged climbing robots have demonstrated linear vertical climbing, with a top speed of 4 cm/s when unloaded. The ACROBOT [21] is capable of climbing vertical and inverted surfaces and can execute turning motions, carrying a load of 0.2 kg at a maximum speed of 0.15 cm/s. A 16-legged climbing robot [25,26] developed at Carnegie Mellon University is able to climb using only two motors and adapt to curved surfaces, with a maximum speed of 4 cm/s while carrying a 2 N load. Despite these advancements, many of these systems overlook the importance of the robot’s operability. While locomotion and load-bearing abilities enhance climbing performance, they do not directly translate into operational functionality or task-specific capabilities. Dry adhesion-based legged robots, due to the repetitive adhesion and detachment cycles required for climbing, have inherently lower load-bearing capacity compared to wheeled or tracked climbing robots. Therefore, an autonomous climbing manipulator arm is the ideal solution, combining with a dry adhesion legged climbing robot to accomplish specific manipulation tasks during work, and climbing autonomously when there are no tasks, to reduce the load pressure on the dry-adhesive legged climbing robot, and to ensure the stable motion of the robot.

To develop climbing robots with enhanced operational capabilities, gripper-based climbing robots offer significant advantages. However, current gripper-based robots are limited to climbing in fixed environments, such as steel pipes, metal frames, or flat surfaces, and lack the ability to perch on arbitrary surfaces or objects. Additionally, they do not possess adaptive grasping capabilities. Inspired by the Fin Ray Effect, flexible grippers, which mimic the deformation of fish fins, were developed. When pressure is applied to one side of the fin, it bends in the opposite direction, enabling a wrapping effect around an object’s surface, allowing for adaptive and compliant grasping. Despite these advantages, flexible grippers based on the Fin Ray Effect currently lack active detachment capabilities, rendering them unsuitable for use as adhesion feet in climbing robots. Even when adhesion materials, such as MST (micro-suction tape), are added to the Fin Ray inspired flexible gripper to ensure sufficient adhesion, the lack of a corresponding detachment mechanism leads to challenges. Grippers can only perform passive, rigid detachment, requiring excessive detachment force, and are unable to achieve flexible edge detachment, which affects the robot’s ability to climb.

To enhance the adaptive grasping capabilities of dry adhesion legged climbing robots, we developed the MST-G, a novel robot capable of autonomous climbing, perching, and adaptive flexible grasping. The MST-G features an innovative design that improves the traditional Fin Ray inspired flexible gripper. By breaking the connection between the longitudinal and transverse beams on the outer side of the gripper and replacing it with two rows of magnets arranged with specific magnetic polarities, we ensure stable adaptive grasping and efficient climbing performance. During adhesion and gripping, the magnetic attraction between the two rows of magnets aligns the longitudinal and transverse beams, maintaining the same functional characteristics as traditional Fin Ray inspired flexible grippers. For detachment, the two rows of magnets are separated by the detachment gear set driving the outer longitudinal and transverse beams of the detachment belt traction, realizing detachment from the tip edge of the flexible gripper. This ensures effective and active detachment during the climbing process and guarantees that the detachment force is minimized. The main contributions of this paper are summarized as follows:

(1) A MST-based gripper climbing robot, MST-G, which is capable of autonomous climbing, perching, and adaptive flexible grasping, is proposed.
(2) An active detachment flexible gripper is proposed, which realizes stable adaptive gripping and active detachment by replacing the traditional fixed connection between the longitudinal and transverse beams of the outer side of the Fin Ray inspired flexible grippers by two rows of magnets aligned according to specific magnetic poles.
(3) A robust controller based on prescribed performance is proposed to limit the joint tracking error to a specified safety bound of 0.002 radians and 0.0001 m, ensuring safe and reliable execution of the robot’s motion trajectory.

## 2. Design of a Grip Climbing Robot MST-G

**Figure 1:**
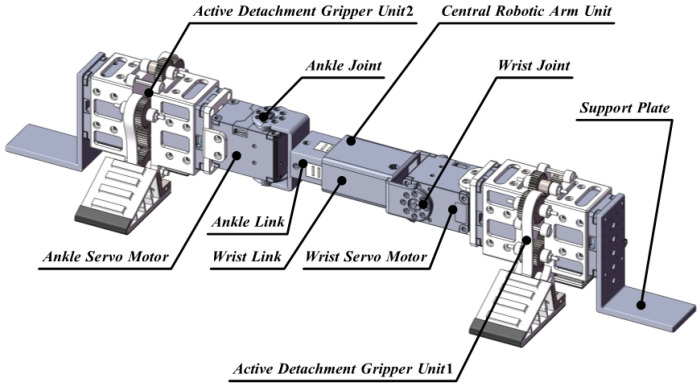
Structure design of MST-G.

To enhance the adaptive grasping capabilities of dry adhesion legged climbing robots, this paper introduces the development of MST-G, a novel dry adhesion climbing robot capable of autonomous climbing, perching, and flexible adaptive grasping. The robot consists of two active detachment units (Unit 1 and Unit 2) and a central robotic arm unit, as shown in Figure 1. Unit 1 comprises a support plate, an active detachment gripper, and a wrist servo motor, while Unit 2 consists of a support plate, an active detachment gripper, and an ankle servo motor. These two units are primarily responsible for adaptive grasping, adhesion, and detachment movements. The critical function of the support plate, which is fixedly attached to the active detachment gripper, is that it resists the overturning moment and remains stable during autonomous climbing.

The central robotic arm unit consists of a wrist link, ankle link, and an embedded linear motor. Wrist link and ankle link are connected to the rotational axes of the wrist and ankle servo motors, respectively, forming a 3 dof arm structure. This configuration provides the necessary spatial degrees of freedom for the flexible gripper to perform adaptive grasping.

The proposed active detachment gripper climbing robot offers two primary functions:

**Autonomous Climbing as a Standalone Legged Robot:** The robot operates independently as a climbing system by leveraging its active detachment grippers to perform rapid adhesion and detachment motions, linear motors provide linear motion to facilitate forward movement, enabling autonomous climbing. In its initial state, both active detachment grippers are adhered to the climbing surface. The Unit 1 begins detachment. Once Unit 1 completes the detachment, the linear motor drives it forward while Unit 2 remains adhesion in place. Upon reaching its desired position, Unit 1 reattaches adaptively to the surface. The gripper in Unit 2 detaches and moves forward via the linear motor. This alternating process enables continuous linear climbing.

**Adaptive Perching and Load Adjustment:** The MST-G can also perch on various objects, including the body of other climbing robots, by using its adaptive grasping capabilities. When perched on a climbing robot, it functions as a flexible adaptive gripper. The central robotic arm provides the necessary spatial degrees of freedom to position the active detachment grippers for targeted grasping of objects.

## 3. Active Detachment Flexible Gripper

The active detachment gripper builds upon the traditional Fin Ray inspired flexible grippers, integrating MST on the gripping surface. The gripper achieves active detachment by detachment the longitudinal and transverse beams of the outer frame using two rows of magnets with a specific magnetic pole arrangement. This configuration allows the MST to detach from the edge of the gripper’s tip. To ensure minimal detachment force, a detailed analysis of the properties of the MST is first required.

### 3.1. Micro Suction Tape Adhesion and Detachment Performance Analysis

MST is a commercial dry adhesive material manufactured by Sewell Direct. It is inexpensive, durable, and can withstand normal adhesion pressures of up to 80 kPa without leaving any residue. MST shows strong durability and can be used repeatedly without losing its holding power. The adhesion model [27] of MST can be expressed as:

(1) $$\sigma_{\mathrm{adhesion}}=\sigma_{\mathrm{vacuum}}+\sigma_{\mathrm{van}}$$

with

(2) $$\left\{\begin{array}{cc} \sigma_{\mathrm{vacuum}} & =1\;\mathrm{atm}\left[1-\frac{\left(1-\frac{\sigma_{\mathrm{preload}}}{E_{\mathrm{effective}}}\right)}{\left(1+\frac{\sigma_{\mathrm{load}}}{E_{\mathrm{effective}}}\right)}\right]\pi R^{2}D\\ \sigma_{\mathrm{van}} & =\left[G\mathrm{lg}\left(\frac{\sigma_{\mathrm{preload}}}{1\;\mathrm{kPa}}\right)+K\right] \end{array}\right.$$

where $\sigma_{\mathrm{adhesion}}$ denotes adhesion stress; 1atm is standard atmospheric pressure; $\sigma_{\mathrm{preload}}$ defines the preload stress; $\sigma_{\mathrm{load}}$ is the external stress to break adhesion; R is the radius of the voids, D denotes the total effective density of the voids; $E_{\mathrm{effective}}$ is the modulus of elasticity of the material; and G and K are experimentally derived constants.

Equation (1) reveals that the adhesion force of MST consists of two constituents, namely, the vacuum force $\sigma_{\mathrm{vacuum}}$ and the van der Waals force $\sigma_{\mathrm{van}}$. MST employs micro-suction cups to generate vacuum force and the rest of the area for van der Waals force. Thus, MST exhibits superior adhesion properties and is an exemplary material for dry adhesive climbing robots.

Additionally, the detachment effect of MST can be analyzed qualitatively using the detachment force equation proposed by Kendall [28] for viscoelastic objects to guide detachment.

(3) $${\left(\frac{F_{d}}{b}\right)}^{2}\frac{1}{2dE}+\left(\frac{F_{d}}{b}\right)\left(1-\cos\theta \right)-R=0$$

where θ is the angle between the adhesive foot and the surface; $F_{d}$ is the detachment force; *d* is the thickness of the adhesion foot; *E* is Young’s modulus of MST; and *R* is the adhesive energy, which was determined experimentally.

From the above formula, the larger the detachment angle, the smaller the detachment force, and the best detachment effect originates from the edge.

### 3.2. Design of Active Detachment Gripper

**Figure 2:**
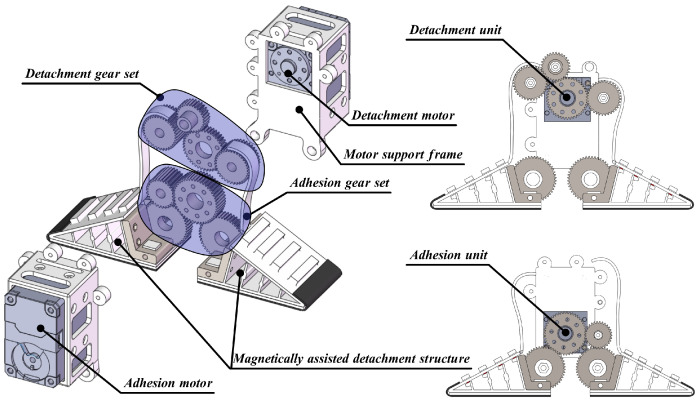
Structure design of the active detachment gripper.

The active detachment gripper consists primarily of a magnetically assisted detachment structure, a detachment unit, and an adhesion unit. The detailed structural diagram and exploded view of the active detachment gripper are shown in Figure 2.

**Figure 3:**
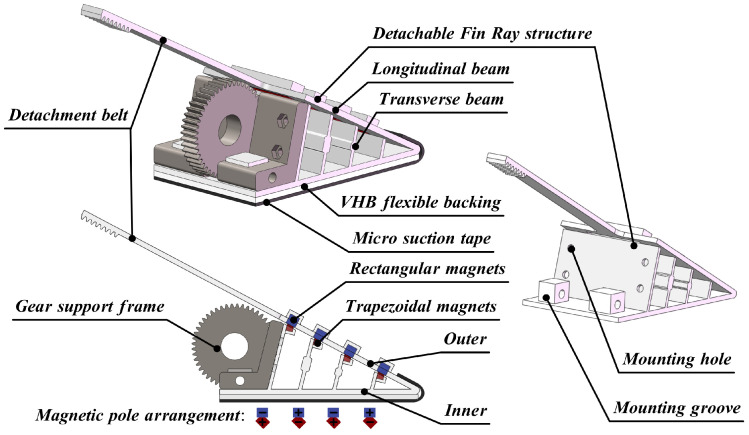
Magnetically assisted detachment structure.

The magnetically assisted detachment structure primarily consists of a detachable Fin Ray structure, detachment belt, trapezoidal magnets, rectangular magnets, gear support frame, VHB flexible backing, and micro-suction tape. The key difference between this detachable Fin Ray structure and existing Fin Ray structure is the connection method: the inner longitudinal and transverse beams of detachable Fin Ray structure are fixedly connected (via flexible hinges), while the outer beams are connected by two rows of magnets with specific polar arrangements. In this way, an active detachment effect is achieved, which ensures that the MST is detached from the edge of the tip of the detachable Fin Ray structure, guaranteeing an optimal detachment force and minimizing the impact on the system. The detailed structure is illustrated in Figure 3. To clarify the contact configuration between the two rows of magnets, the trapezoidal magnets are colored red, and the rectangular magnets are colored blue. Additionally, the magnetic pole arrangement is indicated in the Figure 3, with + representing the positive pole and − representing the negative pole.

Eight magnet slots are designed at the joints between the outer longitudinal and transverse beams of the detachable Fin Ray structure to house the trapezoidal and rectangular magnets. The trapezoidal design of the magnets ensures a tight fit between the magnets on longitudinal and transverse beams. The alternating magnetic pole arrangement between the two rows is intended to prevent magnetic interaction between adjacent magnets, avoiding alignment issues that could result in detachment failure due to misplaced magnetic attraction during detachment or adhesion.

The detachment belt is fixedly connected to the longitudinal beam of the detachable Fin Ray structure and is significantly narrower than the beam itself. It is primarily linked to the detachment gear set. When the detachment motor rotates, it drives the movement of the detachment belt, which in turn moves the longitudinal beam. This motion causes the two rows of magnets with specific polar arrangements to separate, leading to the detachment of the outer longitudinal and transverse beams. As a result, the MST is disengaged from the edge of the Fin Ray structure’s tip. The detachment belt is connected to the detachment gear set, and its narrower width is designed to maximize the distance it can travel on the gear of a fixed thickness. The gear support frame is primarily used to hold the detachable Fin Ray structure and to ensure that it is capable of gripping, adhesion, and detachment motions by using the end of the motor support frame as a center of rotation to transmit the clamping force of the adhesion motor to the detachable Fin Ray structure through engagement with the adhesion gear set. Additionally, to ensure a secure attachment between the detachable Fin Ray structure and the gear support frame, the design incorporates mounting holes and mounting grooves for precise and reliable fixation.

The MST is affixed to the inner surface of the detachable Fin Ray structure using a VHB flexible backing for adhesion. As a dry adhesive, MST assists in both adhesion and gripping tasks. The VHB flexible backing ensures that the adhesive force is distributed evenly across the surface, allowing for reliable adhesion while maintaining the flexibility needed for adaptive adhesion in the Fin Ray structure.

The adhesion unit comprises an adhesion gear set, an adhesion motor, and a motor support frame. The unit ensures effective adaptive gripping and adhesion action by driving the adhesion motor, which rotates the gear support frame.

The detachment unit consists of a detachment gear set, a detachment motor, and a motor support frame. The detachment gear set is fixedly connected to the detachment belt. Its primary function is to drive the detachment belt, facilitating the release of the MST from the edge of the detachable Fin Ray structure, thereby minimizing the detachment force.

#### Detachment and Adhesion Process

**Figure 4:**
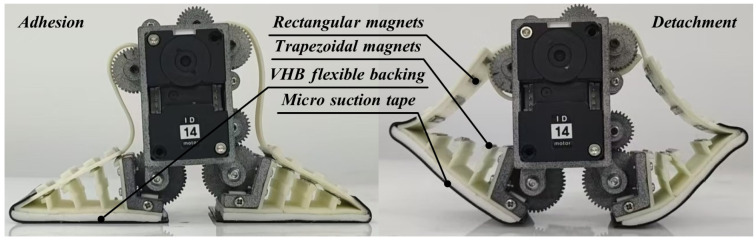
Detachment and adhesion processes of the active detachment gripper.

The detachment and adhesion processes of the active detachment gripper can be categorized into two stages, as shown in Figure 4. Prior to detachment, the adhesion motor and the detachment motor must work in concert to facilitate adaptive adhesion to the surface. This requires a little more movement of the magnetically assisted detachment structure toward the adhesion surface to apply sufficient preload, ensuring that the MST makes adequate contact with the surface. Specific details of these two stages are given below:

**Detachment Motion Stage:** The adhesion motor must maintain its current angle to keep the gripper in an adhesion state. Simultaneously, the detachment motor drives the detachment belt in a tangential motion along the detachment gear set. This action sequentially releases the magnets on the outer longitudinal and transverse beams of the gripper from top to bottom, allowing the MST to detach from the edge of the detachable Fin Ray structure.

**Adaptive Adhesion Stage:** The adhesion motor rotates the magnetically assisted detachment structure to the appropriate position for the current surface (initial position for flat surfaces and corresponding curvature position for curved surfaces). The detachment motor must coordinate with the adhesion motor’s movement to resumption the initial adhesive position, allowing the outer longitudinal and transverse beams to return to their original alignment through the magnetic attraction of the magnets. This action enables the active detachment gripper to achieve the same adaptive enveloping effect as traditional Fin Ray flexible grippers, ensuring that the magnets remain tightly aligned without separation. The adhesion and detachment motors are then driven to move in the same direction to facilitate adaptive adhesion.

## 4. Kinematics and Dynamics Modeling of MST-G

The MST-G requires the development of three distinct models: (1) an adaptive grasping and adhesion model of the active detachment gripper unit, which establishes the relationship between the size of the object being grasped and the gripper’s grasping angle. This model aims to enable adaptive grasping of objects with varying dimensions; (2) a kinematics and dynamics model of MST-G when functioning as a fixed-base auxiliary manipulator. Once MST-G achieves perching on an object or the body of a climbing robot, one of its active detachment grippers becomes fixed, it can be treated as a three-degree-of-freedom fixed-base robotic arm. To achieve trajectory motion and trajectory tracking control in this configuration, it is essential to establish the forward kinematics, inverse kinematics, and dynamics of MST-G as a fixed-base manipulator; and (3) a climbing model when the robot performs autonomous climbing. However, since MST-G relies solely on the central linear motor and the adaptive adhesion motions of the active detachment gripper units during autonomous climbing, only the fixed-base manipulator model and the adaptive grasping and adhesion model need to be established.

### 4.1. Adaptive Grasping and Adhesion Model

**Figure 5:**
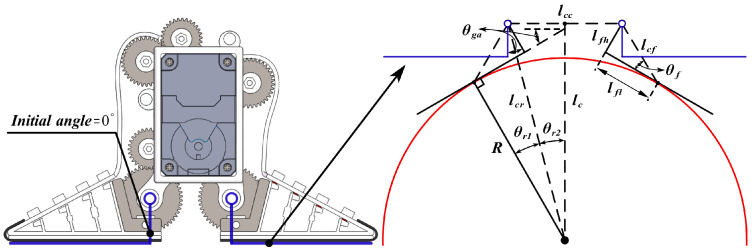
Simplified front view of active detachment gripper.

To realize the adhesion and gripping motions, the adhesion and gripping angles $\theta_{ga}$ of the active detachment gripper need to be given. For gripping flat objects and adhesion planes, it is sufficient to keep the gripper angle at the initial angle of zero position. For grasping curved objects and adherent curves, it is necessary to build an adaptive grasping and adhesion model by knowing the arc radius *R* of the adhesion curve and the object to be grasped, so as to obtain the adhesion and grasping angles $\theta_{ga}$ of the active detachment gripper. The front view of the active detachment gripper is simplified for easy representation as shown in Figure 5.

The Euclidean distance $l_{cf}$ between the rotation center of the gear support frame and the contact point of the detachable Fin Ray structure with the adhered surface, along with the corresponding angle $\theta_{f}$, can be directly obtained.

(4) $$\left\{\begin{array}{c} l_{cf}=\sqrt{l_{fh}^{2}+l_{fl}^{2}}\\ \theta_{f}=\mathrm{atan}2\left(l_{fh},l_{fl}\right) \end{array}\right.$$

where $l_{fh}$ represents the lateral distance between the contact point of the detachable Fin Ray structure with the adhered surface and the rotation center of the gear support frame; and $l_{fl}$ denotes the vertical distance between the contact point of the detachable Fin Ray structure with the adhered surface and the rotation center of the gear support frame. For effective adhesion and grasping, it is preferable that the contact point remains fixed. Therefore, $l_{fl}$ remains relatively constant.

By the cosine theorem, one obtains:

(5) $$\left\{\begin{array}{c} l_{cr}=\sqrt{l_{cf}^{2}+R^{2}-2l_{cf}R\cos\left(\theta_{f}+\frac{\pi }{2}\right)}\\ \theta_{r1}=\arccos\frac{R^{2}+l_{cr}^{2}-l_{cf}^{2}}{2Rl_{cr}} \end{array}\right.$$

where $l_{cr}$ represents the distance between the rotation center of the gear support frame and the center of the circular arc of the adhered surface. *R* represents the radius of the arc corresponding to the gripped or adhered surface.

Through Equation (5) and the Pythagorean Theorem, one obtains:

(6) $$\left\{\begin{array}{c} l_{c}=\sqrt{l_{cr}^{2}-{\frac{l_{cc}}{2}}^{2}}\\ \theta_{r2}=\arcsin\frac{l_{cc}}{2l_{cr}} \end{array}\right.$$

where $l_{c}$ is the distance between the rotation centers of the gear support frame and the arc center of the adhered surface. $l_{cc}$ represents the fixed distance between the two gear support frame rotation centers at the gripper.

Thus, the adaptive grasping and adhesion angle $\theta_{ga}$ can be calculated as:

(7) $$\theta_{ga}=\theta_{r1}+\theta_{r2}$$

### 4.2. Forward Kinematic Modeling of Fixed Base Manipulator

**Figure 6:**
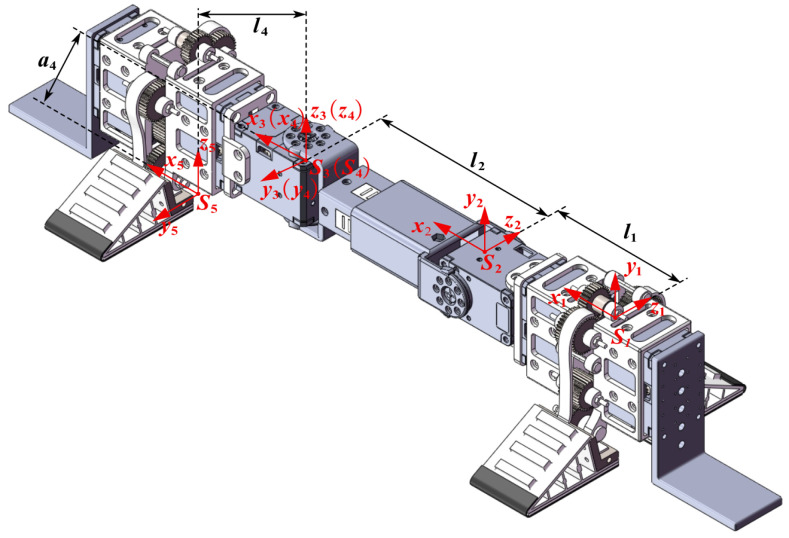
Position in the coordinate frame of the joints of the active detachable gripper.

We establish the forward kinematics model for the active detachment gripper climbing robot when used as an auxiliary manipulator. Assuming that the active detachment gripper Unit 1 is used as a fixed gripper and the active detachment gripper Unit 2 is used as an adhesive gripper, forming a structure similar to a fixed-base robotic arm. A base coordinate frame $S_{1}$ is defined at the center of mass of gripper Unit 1. Coordinate frames are then established at the wrist joint $S_{2}$, the ankle joint $S_{3},S_{4}$, and the rotational center of the two gear support frame of the adhesive gripper $S_{5}$, as shown in Figure 6. The corresponding Denavit-Hartenberg (D-H) kinematic parameters and joint variables are listed in Table 1.

Using Table 1, the corresponding homogeneous transformation matrix ${\,}_{i}^{i+1}T$ is derived.

(8) $${\,}_{2}^{1}T=\left(\begin{array}{cccc} \cos\theta_{1} & -\sin\theta_{1} & 0 & l_{1}\\ \sin\theta_{1} & \cos\theta_{1} & 0 & 0\\ 0 & 0 & 1 & 0\\ 0 & 0 & 0 & 1 \end{array}\right)$$

(9) $${\,}_{3}^{2}T=\left(\begin{array}{cccc} 1 & 0 & 0 & l_{2}\\ 0 & 0 & 1 & 0\\ 0 & -1 & 0 & 0\\ 0 & 0 & 0 & 1 \end{array}\right)$$

(10) $${\,}_{4}^{3}T=\left(\begin{array}{cccc} \cos\theta_{3} & -\sin\theta_{3} & 0 & 0\\ \sin\theta_{3} & \cos\theta_{3} & 0 & 0\\ 0 & 0 & 1 & 0\\ 0 & 0 & 0 & 1 \end{array}\right)$$

(11) $${\,}_{5}^{4}T=\left(\begin{array}{cccc} 1 & 0 & 0 & l_{4}\\ 0 & 1 & 0 & 0\\ 0 & 0 & 1 & -a_{4}\\ 0 & 0 & 0 & 1 \end{array}\right)$$

Ultimately, the homogeneous transformation matrix of the forward kinematics for the climbing robot as a fixed-base manipulator can be obtained:

(12) $${\,}_{5}^{1}T={\,}_{2}^{1}T{\,}_{3}^{2}T{\,}_{4}^{3}T{\,}_{5}^{4}T=\left(\begin{array}{cccc} c_{1}c_{3} & -c_{1}s_{3} & -s_{1} & l_{4}c_{1}c_{3}+a_{4}s_{1}+l_{2}c_{1}+l_{1}\\ s_{1}c_{3} & -s_{1}s_{3} & c_{1} & l_{4}s_{1}c_{3}-a_{4}c_{1}+l_{2}s_{1}\\ -s_{3} & -c_{3} & 0 & -l_{4}s_{3}\\ 0 & 0 & 0 & 1 \end{array}\right)$$

where $s_{1}$ represents $\sin\theta_{1}$, and $c_{1}$ represents $\cos\theta_{1}$, with other symbols following similar notation.

### 4.3. Inverse Kinematic Modeling of Fixed Base Manipulator

Using the ${\,}_{2}^{1}T^{-1}$ left-multiplication of the forward kinematics equation ${\,}_{5}^{1}T$, we obtain:

(13) $${\,}_{2}^{1}T^{-1}{\,}_{5}^{1}T={\,}_{3}^{2}T{\,}_{4}^{3}T{\,}_{5}^{4}T{=}_{5}^{2}T$$

(14) $$\begin{array}{cc} {\,}_{2}^{1}T^{-1}{\,}_{5}^{1}T & =\left(\begin{array}{cccc} \cos\theta_{1} & \sin\theta_{1} & 0 & -l_{1}\cos\theta_{1}\\ -\sin\theta_{1} & \cos\theta_{1} & 0 & l_{1}\sin\theta_{1}\\ 0 & 0 & 1 & 0\\ 0 & 0 & 0 & 1 \end{array}\right)\left(\begin{array}{cccc} n_{x} & o_{x} & a_{x} & p_{x}\\ n_{y} & o_{y} & a_{y} & p_{y}\\ n_{z} & o_{z} & a_{z} & p_{z}\\ 0 & 0 & 0 & 1 \end{array}\right)\\ \; & =\left(\begin{array}{cccc} f_{\left(1,1\right)} & f_{\left(1,2\right)} & f_{\left(1,3\right)} & f_{\left(1,4\right)}\\ f_{\left(2,1\right)} & f_{\left(2,2\right)} & f_{\left(2,3\right)} & f_{\left(2,4\right)}\\ f_{\left(3,1\right)} & f_{\left(3,2\right)} & f_{\left(3,3\right)} & f_{\left(3,4\right)}\\ 0 & 0 & 0 & 1 \end{array}\right) \end{array}$$

with

(15) $$\left\{\begin{array}{c} f_{\left(1,4\right)}=p_{x}\cos\theta_{1}-l_{1}\cos\theta_{1}+p_{y}\sin\theta_{1}\\ f_{\left(2,4\right)}=p_{y}\cos\theta_{1}+l_{1}\sin\theta_{1}-p_{x}\sin\theta_{1}\\ f_{\left(3,4\right)}=p_{z} \end{array}\right.$$

where the position of the adhesion gripper relative to the base frame $S_{1}$ in space is $\left(p_{x},p_{y},p_{z}\right)$, and the orientation of the gripper relative to $S_{1}$ can be represented by the rotation matrix components $n_{x}$, $o_{x}$, $a_{x}$, $n_{y}$, $o_{y}$, $a_{y}$, $n_{z}$, $o_{z}$, and $a_{z}$.

Additionally, the transformation matrix ${\,}_{5}^{2}T$ is given as:

(16) $${\,}_{5}^{2}T={\,}_{3}^{2}T{\,}_{4}^{3}T{\,}_{5}^{4}T=\left(\begin{array}{cccc} \cos\theta_{3} & -\sin\theta_{3} & 0 & l_{2}+l_{4}\cos\theta_{3}\\ 0 & 0 & 1 & -a_{4}\\ -\sin\theta_{3} & -\cos\theta_{3} & 0 & -l_{4}\sin\theta_{3}\\ 0 & 0 & 0 & 1 \end{array}\right)$$

By setting $f_{\left(3,4\right)}=-l_{4}\sin\theta_{3}$:

(17) $$\theta_{3}=\arcsin\frac{-p_{z}}{l_{4}}$$

Similarly, setting $f_{\left(2,4\right)}=-a_{4}$ yields:

(18) $$\theta_{1}=\mathrm{atan}2\left(p_{y},p_{x}-l_{1}\right)-\mathrm{atan}2\left(-a_{4},\pm \sqrt{{\left(p_{x}-l_{1}\right)}^{2}+p_{y}^{2}-a_{4}^{2}}\right)$$

And the constraint range of $\theta_{1}$ is $\left(-\frac{\pi }{2},\frac{\pi }{2}\right)$. Values outside this range are discarded to obtain the true angle of the wrist joint $\theta_{1}$.

(19) $$\theta_{1}=\mathrm{atan}2\left(p_{y},p_{x}-l_{1}\right)-\mathrm{atan}2\left(-a_{4},\sqrt{{\left(p_{x}-l_{1}\right)}^{2}+p_{y}^{2}-a_{4}^{2}}\right)$$

Setting $f_{\left(1,4\right)}=l_{2}+l_{4}\cos\theta_{3}$ yields.

(20) $$l_{2}=p_{x}\cos\theta_{1}-l_{1}\cos\theta_{1}+p_{y}\sin\theta_{1}-l_{4}\cos\theta_{3}$$

The final inverse kinematic model of the fixed-base manipulator is obtained, which can convert the running trajectory of the robot’s end to the corresponding joint motion trajectory for subsequent control.

### 4.4. Dynamic Modeling of Fixed Base Manipulator

In this paper, the Lagrange equation is used to model the inverse dynamics in the form of Equation (21).

(21) $$\left\{\begin{array}{c} L=K-P\\ \frac{d}{dt}\left(\frac{\partial L}{\partial \dot{\theta }}\right)-\frac{\partial L}{\partial \theta }=\tau \end{array}\right.$$

where *K* is the total kinetic energy; *P* is the total potential energy; *L* represents the Lagrangian function; θ represents the joint angle; $\dot{\theta }$ represents the joint angular velocity; and τ represents the corresponding joint force distance.

In order to obtain a dynamic model of an active detachment gripper climbing robot with a fixed base, it is necessary to obtain the angular velocities of the center of mass of each linkage with respect to $S_{1}$, and in this paper, we stipulate that the counterclockwise rotation direction is positive. According to the principle of rigid body angular velocity superposition, the angular velocities of different rigid bodies can be freely added when expressed in the same coordinate system, allowing us to obtain the desired rigid body angular velocities relative to the fixed world frame. Based on this principle, the angular velocities of each rigid body with respect to the world frame can be derived.

(22) $$\left\{\begin{array}{c} \omega_{w}={\dot{\theta }}_{1}\\ \omega_{a}={\dot{\theta }}_{1}\\ \omega_{f}={\dot{\theta }}_{1}+{\dot{\theta }}_{2} \end{array}\right.$$

where $\omega_{w}$ represents the joint angular velocity at the wrist; $\omega_{a}$ represents the linear velocity; and $\omega_{f}$ represents the joint angular velocity at the ankle.

The kinetic energy $K_{w}$ and potential energy $P_{w}$ of the wrist link are expressed as:

(23) $$\left\{\begin{array}{c} K_{w}=\frac{1}{2}m_{w}l_{w}^{2}{\dot{\theta }}_{1}^{2}+\frac{1}{2}J_{w}\omega_{w}^{2}\\ P_{w}=m_{w}gl_{w}\sin\theta_{1} \end{array}\right.$$

where $m_{w}$ is the mass of the wrist link, $l_{w}$ is the Euclidean distance between the center of mass of the wrist link and the wrist joint, and $J_{w}$ is the moment of inertia of the wrist link’s center of mass about the z-axis.

The kinetic energy $K_{a}$ and potential energy $P_{a}$ of the ankle link are given by:

(24) $$\left\{\begin{array}{c} K_{a}=\frac{1}{2}m_{a}\left({\left(l_{2}-l_{a}\right)}^{2}{\dot{\theta }}_{1}^{2}+{\dot{l_{2}}}^{2}\right)+\frac{1}{2}J_{a}\omega_{a}^{2}\\ P_{a}=m_{a}g\left(l_{2}-l_{a}\right)\sin\theta_{1} \end{array}\right.$$

where $m_{a}$ is the mass of the ankle link; $l_{a}$ is the Euclidean distance between the center of mass of the ankle link and the ankle joint; and $J_{a}$ is the moment of inertia of the ankle link’s center of mass about the z-axis.

The kinetic energy $K_{f}$ and potential energy $P_{f}$ of the adhesive gripper link are:

(25) $$\left\{\begin{array}{c} K_{f}=\frac{1}{2}m_{f}v_{f}^{2}+\frac{1}{2}J_{f}\omega_{f}^{2}\\ P_{f}=m_{f}g\left(l_{2}+l_{4}\cos\theta_{3}\right)\sin\theta_{1} \end{array}\right.$$

with

(26) $$\left\{\begin{array}{c} v_{f}^{2}={\dot{x}}_{f}^{2}+{\dot{y}}_{f}^{2}+{\dot{z}}_{f}^{2}\\ x_{f}=l_{2}+l_{4}\cos\theta_{3}\\ y_{f}=\left(l_{2}+l_{4}\cos\left(\theta_{3}\right)\right)\sin\theta_{1}\\ z_{f}=l_{4}\sin\theta_{3} \end{array}\right.$$

where $m_{f}$ is the mass of the adhesive gripper, and $J_{f}$ is the moment of inertia of the adhesive gripper’s center of mass about the z-axis.

Combining Equations (23)–(25) gives.

(27) L=K−P

with

(28) $$\left\{\begin{array}{c} K=K_{w}+K_{a}+K_{f}\\ P=P_{w}+P_{a}+P_{f} \end{array}\right.$$

Substituting Equation (27) into (21) yields.

(29) $$\tau_{d}=M\ddot{\theta }+C+G+D$$

with

(30) $$\left\{\begin{array}{c} M=\left[\begin{array}{ccc} m_{w}l_{w}^{2}+m_{a}{\left(l_{2}-l_{a}\right)}^{2}+J_{w}+J_{a}+J_{f} & 0 & 0\\ 0 & m_{a}+m_{f} & 0\\ 0 & 0 & J_{f} \end{array}\right]\\ C=\left[\begin{array}{c} 2m_{a}\left(l_{2}-l_{a}\right){\dot{\theta }}_{1}\\ -m_{a}\left(l_{2}-l_{a}\right){\dot{\theta }}_{1}^{2}\\ m_{f}l_{4}{\dot{l}}_{2}\cos\theta_{3} \end{array}\right]\\ G=\left[\begin{array}{c} m_{w}l_{w}g\cos\theta_{1}+m_{a}\left(l_{2}-l_{a}\right)g\cos\theta_{1}+m_{f}\left(l_{2}+l_{4}\cos\theta_{3}\right)g\cos\theta_{1}\\ \left(m_{a}+m_{f}\right)g\sin\theta_{1}\\ -m_{f}l_{4}g\sin\theta_{1}\sin\theta_{3} \end{array}\right] \end{array}\right.$$

where the matrix $M\in R^{n\times n}$ is the inertia matrix of the robot, the matrix $C\in R^{n\times n}$ is the robot’s Coriolis and centrifugal force matrix, and $G\in R^{n\times n}$ is the robot’s gravity matrix, *D* is the interference matrix of the system.

Finally, the dynamic equations are obtained using the Lagrangian equation.

## 5. Robust Controller Based on Prescribed Performance

The structure design of the active detachment gripper enables adaptive adhesion and gripping, thus reducing the need for high control precision. Simply adjusting the gripping angle according to the curvature or the radius of the object is sufficient. The autonomous climbing of the gripper climbing robot MST-G is satisfied by controlling only the movement of the linear motors while ensuring adaptive adhesion and grasping. However, when the MST-G functions as a fixed base manipulator, its performance in spatial trajectory tracking directly impacts the success of object grasping, necessitating a precise tracking control algorithm. Therefore, this paper proposes a robust controller based on prescribed performance to ensure stable mobile grasping operations.

This paper proposes a robust controller based on prescribed performance. Utilizing the dynamics of the fixed-base active detachment gripper climbing robot, joint position error bounds are prescribed. Through error transformation techniques, the constrained error bounds are converted into unconstrained equivalent errors, ensuring that the joint position errors remain within the defined limits. Additionally, a sliding mode controller compensates for the unknown disturbances caused by grasping motions, achieving robust control performance and ensuring stable grasping operations for the robot. Considering the performance of the actual dynamical system of the robot and the subsequent stability proof, the following lemmas and assumptions are defined.

**Lemma** **1.** *The inertia matrix M in the dynamic equations of an the active detachment gripper climbing robot (29) as a fixed base is symmetric and positive definite. Therefore, $M^{-1}$ is bounded.*

**Assumption** **1.** *The joint angles $\theta_{i}$ and joint angular velocities $\omega_{i}$ in the kinetic equations of the system (29) are bounded and measurable.*

**Assumption** **2.** *If $\|\theta_{i}\|$ and $\|\omega_{i}\|$ are bounded, then ‖C‖ and ‖G‖ are bounded.*

For a fixed-base active detachment gripper climbing robot (29) dynamics system, the control goal is that the joint trajectory $\theta_{i}$ tracks a given reference joint trajectory $\theta_{id}$, and for ease of representation make $\theta_{2}=l_{2}$ and $\omega_{2}=\dot{l_{2}}$. Then the corresponding joint angle tracking error and joint angular velocity tracking error can be expressed as:

(31) $$\left\{\begin{array}{c} e_{i}=\theta_{id}-\theta_{i}\\ \delta_{i}=\omega_{id}-\omega_{i},\;\;i=1,2,3. \end{array}\right.$$

where $\omega_{id}$ is the virtual control quantity.

To keep the error within the specified bounds, an exponential prescribed performance function $\rho_{i}\left(t\right)=\left(\rho_{i0}-\rho_{i\infty }\right)\exp\left(-lt\right)+\rho_{i\infty }$ and an exponential error transformation function $S\left(z_{i}\right)=\frac{e_{i}^{z_{i}}-e_{i}^{-z_{i}}}{e_{i}^{z_{i}}+e_{i}^{-z_{i}}}$ are introduced, where $\rho_{i0}>0$ is the initial value of the prescribed performance function, $\rho_{i\infty }>0$ is the final value of the prescribed performance function, and $\rho_{i\infty }<\rho_{i0}$.

A new state variable is defined as $\alpha_{i}=\frac{e_{i}\left(t\right)}{\rho_{i}\left(t\right)}$, with the equivalent unconstrained error $z_{i}\left(t\right)$ expressed as:

(32) $$z_{i}\left(t\right)=S^{-1}\left(\frac{e_{i}\left(t\right)}{\rho_{i}\left(t\right)}\right)=S^{-1}\left(\alpha_{i}\right)=\frac{1}{2}\ln\left(\frac{1+\alpha_{i}}{1-\alpha_{i}}\right),\;\;i=1,2,3$$

Then, the equivalent error $z_{i}\left(t\right)$ is obtained by derivation for time *t*:

(33) $${\dot{z}}_{i}\left(t\right)=\frac{\dot{\rho_{i}}e_{i}-\rho_{i}\dot{e_{i}}}{e_{i}^{2}-\rho_{i}^{2}},\;\;i=1,2,3$$

To ensure system stability, a positive definite Lyapunov function $V_{1}$ is designed:

(34) $$V_{1}=\frac{1}{2}z^{T}z$$

Differentiating (34) by using (33) and (31) yields:

(35) $$\begin{array}{cc} {\dot{V}}_{1} & =z^{T}\dot{z}=\sum_{i=1}^{3}z_{i}\;\frac{\dot{\rho_{i}}e_{i}-\rho_{i}\dot{e_{i}}}{e_{i}^{2}-\rho_{i}^{2}}\\ \; & =\sum_{i=1}^{3}z_{i}\;\frac{\dot{\rho_{i}}e_{i}-\rho \left(\omega_{id}-\omega_{i}\right)}{e_{i}^{2}-\rho_{i}^{2}} \end{array}$$

with

(36) $$z=\left[\begin{array}{c} z_{1}\\ z_{2}\\ z_{3} \end{array}\right],\;\;\dot{z}=\left[\begin{array}{c} {\dot{z}}_{1}\\ {\dot{z}}_{2}\\ {\dot{z}}_{3} \end{array}\right]$$

For simplification, let $z_{i}\;\frac{1}{e_{i}^{2}-\rho_{i}^{2}}=\gamma_{i}$.

Let the virtual control input $\omega_{id}$ to the following form:

(37) $$\omega_{id}={\dot{\theta }}_{id}-\beta_{i},\;\;i=1,2,3$$

with

(38) $$\beta_{i}=\frac{\dot{\rho_{i}}\gamma_{i}e_{i}+k_{i}e_{i}^{2}}{\rho_{i}\gamma_{i}},\;\;i=1,2,3$$

where $k_{i}>0,\;\;i=1,2,3$.

Inserting Equation (37) into (31) leads to:

(39) $$\omega_{i}=\omega_{id}-\beta_{i}-\delta_{i},\;\;i=1,2,3$$

Substituting (39) into (35) yields:

(40) $$\begin{array}{cc} {\dot{V}}_{1} & =\gamma^{T}\dot{\rho_{i}}e-\gamma \rho_{i}\left(\omega_{d}-\omega \right) \end{array}$$

with

(41) $$\rho =\left[\begin{array}{ccc} \rho_{1} & 0 & 0\\ 0 & \rho_{2} & 0\\ 0 & 0 & \rho_{3} \end{array}\right],\;\;\gamma =\left[\begin{array}{c} \gamma_{1}\\ \gamma_{2}\\ \gamma_{3} \end{array}\right],\;\;\omega_{d}=\left[\begin{array}{c} \omega_{1d}\\ \omega_{2d}\\ \omega_{3d} \end{array}\right],\;\;\omega =\left[\begin{array}{c} \omega_{1}\\ \omega_{2}\\ \omega_{3} \end{array}\right]$$

Incorporating (37) and (39) into (40) yields:

(42) $$\begin{array}{cc} {\dot{V}}_{1} & =-e^{T}ke\leq 0 \end{array}$$

with

(43) $$k=\left[\begin{array}{ccc} k_{1} & 0 & 0\\ 0 & k_{2} & 0\\ 0 & 0 & k_{3} \end{array}\right],\;\;e=\left[\begin{array}{c} e_{1}\\ e_{2}\\ e_{3} \end{array}\right]$$

To handle system uncertainties and disturbances *d*, a sliding mode control algorithm is introduced. First, define the sliding surface function $s_{i}$:

(44) $$s_{i}=\xi_{i}e_{i}+\delta_{i},\;\;i=1,2,3$$

where $\xi_{i}>0,\;\;i=1,2,3$.

A sliding mode reaching law is then designed to ensure the system converges to the sliding surface $s_{i}$:

(45) $${\dot{s}}_{i}=-\eta_{i}\;\mathrm{sign}\left(s_{i}\right)-\sigma_{i}s_{i}+d_{ic}-d_{i},\;\;i=1,2,3$$

with

(46) $$\left\{\begin{array}{cc} d_{ic} & =d_{iu}-d_{il}\mathrm{sign}\left(s_{i}\right)\\ d_{iu} & =\frac{d_{iU}-d_{iL}}{2}\\ d_{il} & =\frac{d_{iU}+d_{iL}}{2},\;\;i=1,2,3 \end{array}\right.$$

where $d_{ic}$ is chosen to guarantee the arrival condition, $|d_{i}|<\eta_{i}$ and $\sigma_{i}>0$, $d_{iU}$ is the upper limit of the system interference and $d_{iL}$ is the lower limit of the system interference.

A positive definite Lyapunov function $V_{2}$ is designed.

(47) $$V_{2}=V_{1}+\frac{1}{2}s^{T}s$$

with

(48) $$s=\left[\begin{array}{c} s_{1}\\ s_{2}\\ s_{3} \end{array}\right]$$

Differentiating (47) by using (40) yields:

(49) $${\dot{V}}_{2}=\gamma^{T}\dot{\rho }e-\gamma^{T}\rho \left(\omega_{d}-\omega \right)+s^{T}\dot{s}$$

Incorporating (37), (39) and (44) into (49) yields:

(50) $${\dot{V}}_{2}=-e^{T}ke+\gamma^{T}\rho \xi e+s^{T}\left(\xi \dot{e}-\rho \gamma +\dot{\delta }\right)$$

Substituting (29), (31), (37) and (39) into (50) yields:

(51) $${\dot{V}}_{2}=-e^{T}ke+\gamma^{T}\rho \xi e+s^{T}\left(\xi \dot{e}-\rho \gamma +\left({\ddot{\theta }}_{d}-\dot{\beta }-\dot{\omega }\right)\right)$$

with

(52) $$\xi =\left[\begin{array}{ccc} \xi_{1} & 0 & 0\\ 0 & \xi_{2} & 0\\ 0 & 0 & \xi_{3} \end{array}\right],\;\;{\ddot{\theta }}_{d}=\left[\begin{array}{c} {\ddot{\theta }}_{1d}\\ {\ddot{\theta }}_{2d}\\ {\ddot{\theta }}_{3d} \end{array}\right]\dot{\beta }=\left[\begin{array}{c} {\dot{\beta }}_{1}\\ {\dot{\beta }}_{2}\\ {\dot{\beta }}_{3} \end{array}\right],\;\;\dot{\omega }=M^{-1}\left(\tau_{d}-C-G-D\right)$$

Let the final control rate τ take the following form:

(53) $$\tau =M(\xi \dot{e}-\rho \gamma +{\ddot{\theta }}_{d}-\dot{\beta }+\eta \;\mathrm{sign}\left(s\right)+\sigma s-d_{c})+C+G$$

with

(54) $$\sigma =\left[\begin{array}{ccc} \sigma_{1} & 0 & 0\\ 0 & \sigma_{2} & 0\\ 0 & 0 & \sigma_{3} \end{array}\right],\;\;\eta =\left[\begin{array}{ccc} \eta_{1} & 0 & 0\\ 0 & \eta_{2} & 0\\ 0 & 0 & \eta_{3} \end{array}\right],\;\;d_{c}=\left[\begin{array}{c} d_{1c}\\ d_{2c}\\ d_{3c} \end{array}\right],\;\;D_{d}=\left[\begin{array}{c} d_{1}\\ d_{2}\\ d_{3} \end{array}\right]=M^{-1}D$$

**Theorem** **1.** *Consider a robotic system (29) with control laws (53). Then, the tracking error converges to the sliding surface and is restricted to the surface for all subsequent time.*

**Proof.**  Consider the following Lyapunov function $V_{2}$. Taking (29) and (53) into (51) gives:

(55) $${\dot{V}}_{2}=-e^{T}ke+\xi \rho {\left(\gamma e\right)}^{T}+s^{T}\dot{s}$$

From (31), we have

(56) $$-e^{T}ke\leq 0$$

From (31), we analyze $\xi \rho {\left(\gamma e\right)}^{T}$ as follows:

(57) $$\left\{\begin{array}{c} \xi \rho {\left(\gamma e\right)}^{T}=0,\;\;\mathrm{when}\;\;e_{i}=0,\;\;i=1,2,3\\ \xi \rho {\left(\gamma e\right)}^{T}<0,\;\;\mathrm{when}\;\;e_{i}>0,\gamma_{i}<0,\rho_{i}>0,and\;\;\xi_{i}>0,\;\;i=1,2,3\\ \xi \rho {\left(\gamma e\right)}^{T}<0,\;\;\mathrm{when}\;\;e_{i}<0,\gamma_{i}>0,\rho_{i}>0,and\;\;\xi_{i}>0,\;\;i=1,2,3 \end{array}\right.$$

According to (44) and (45), it is clear that

(58) $$s^{T}\dot{s}\leq 0$$

Combining Equations (56)–(58), the control rate of Equation (53) satisfies the stability requirements of the controlled system.

(59) $${\dot{V}}_{2}=-e^{T}ke+\xi \rho {\left(\gamma e\right)}^{T}+s^{T}\dot{s}\leq 0$$

The function ${\dot{V}}_{2}$ is negative semi definite and vanished if and only if $e=\left[\begin{array}{c} 0\\ 0\\ 0 \end{array}\right]$ and $\delta =\left[\begin{array}{c} 0\\ 0\\ 0 \end{array}\right]$. By applying the Lasalle theorem [29], the theorem is proved. This implies the tracking error *e* and δ will also converge asymptotically to zero. □

## 6. Experimental Results

### 6.1. Control Algorithm Validation Simulation Experiments for Fixed Base Manipulators

**Figure 7:**
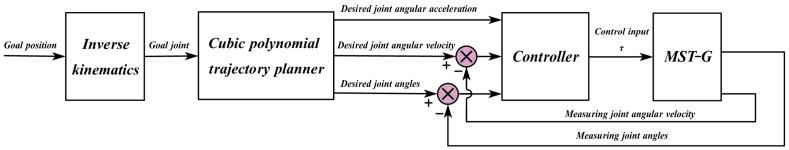
Control flowchart.

**Figure 8:**
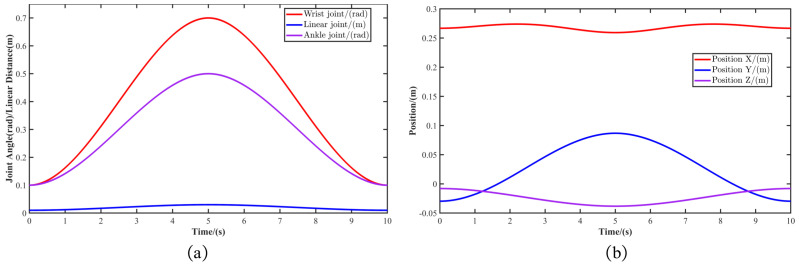
Motion trajectories. (**a**) Motion trajectories planned in joint space. (**b**) Corresponding motion trajectories in Cartesian space.

To validate the control performance of the MST-G gripper climbing robot when functioning as a fixed base manipulator during mobile operations, we designed a trajectory from the start point to the end point and back to the start as a way to observe the tracking effect of the controller. This trajectory first requires the specification of the start and end positions of the gripper’s end effector in Cartesian space. The corresponding joint angles for the start and end points are obtained through the inverse kinematics of the fixed base manipulator (Equations (17), (19) and (20)). The trajectory of MST-G in joint space is then planned using a cubic polynomial. The forward kinematics model of the fixed base manipulator (Equation (12)) is also used to derive the corresponding Cartesian space trajectory. Furthermore, to validate the controller’s performance, the start point was set at a fixed position rather than the initial position, ensuring that the controller’s step tracking performance could be tested. The final start point in Cartesian space is $p_{x}=0.2668\;m$, $p_{y}=-0.0297\;m$, $p_{z}=-0.0080\;m$, and the end point is $p_{x}=0.2593\;m$, $p_{y}=-0.0868\;m$, $p_{z}=-0.0382\;m$. In joint space, the start point is $\theta_{1}=0.1\;\mathrm{rad}$, $l_{2}=0.01\;m$, $\theta_{3}=0.1\;\mathrm{rad}$, and the end point is $\theta_{1}=0.7\;\mathrm{rad}$, $l_{2}=0.03\;m$, $\theta_{3}=0.5\;\mathrm{rad}$. The specific flowchart is shown in Figure 7 and the resulting curves are shown in Figure 8.

**Figure 9:**
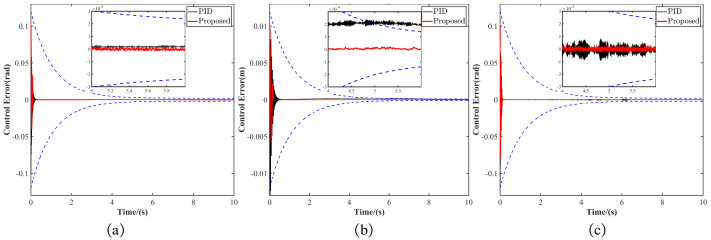
Performance comparison of the robust controller based on Prescribed Performance with PID controller. (**a**) Wrist joint rotation error. (**b**) Linear joint movement error. (**c**) Ankle joint rotation error.

To ensure the tracking performance of MST-G, the initial prescribed performance bounds for the wrist joint were set at $\rho_{10}=0.12\;\mathrm{rad}$, with a final bound of $\rho_{1\infty }=0.002\;\mathrm{rad}$. For the linear joint, the initial prescribed performance bound was $\rho_{20}=0.012\;m$, and the final bound was $\rho_{2\infty }=0.0001\;m$. The wrist joint initial performance bound was $\rho_{30}=0.12\;\mathrm{rad}$, with a final bound of $\rho_{3\infty }=0.002\;\mathrm{rad}$. To highlight the advantages of the robust controller based on Prescribed Performance, a performance comparison between the robust controller based on Prescribed Performance and a traditional PID controller was conducted using the obtained motion trajectory. The results of the control experiments are shown in Figure 9.

As shown in Figure 9a, for wrist joint trajectory tracking, the robust controller based on Prescribed Performance exhibits a shorter settling time and smaller steady-state error. Figure 9b demonstrates that for linear joint trajectory tracking, the proposed controller also achieves a shorter settling time and smaller steady-state error. Notably, around 5 s, the PID controller exceeds the prescribed bounds, whereas the proposed controller remains within the prescribed limits, ensuring safe and stable movement of the manipulator. This is due to the limited regulation capability of the PID controller, as MST-G experiences a velocity reduction to zero and then an increase when moving from the endpoint back to the start. In Figure 9c, for ankle joint trajectory tracking, the proposed controller again shows a shorter settling time and smaller steady-state error. Therefore, the proposed controller guarantees that the tracking error consistently stays within the prescribed limits, ensuring the safe and stable movement of the manipulator.

### 6.2. Adaptive Grasping Experiments

**Figure 10:**
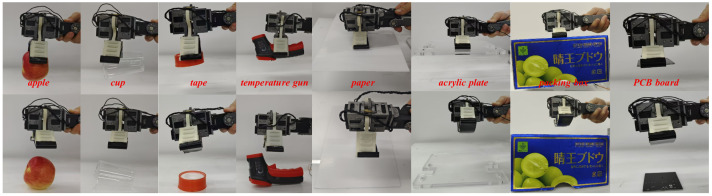
Adaptive grasping experiments for irregular and flat objects in life.

This experiment focuses on validating the adaptive grasping capabilities of MST-G using various irregularly shaped and difficult-to-grasp flat objects encountered in everyday life. When grasping, the grasping angle is obtained according to the adaptive grasping and adhesion model to realize adaptive grasping of different objects, and the specific grasping experiments are shown in Figure 10.

In the adaptive grasping experiments, we tested the system by grasping irregular objects such as an apple, a cup, tape, and a temperature gun, demonstrating the superiority of the active detachment gripper. The gripper’s combination of MST adhesion and adaptive clamping force ensured a high success rate when grasping irregular objects.

For flat objects, which are notoriously challenging due to their inability to be effectively enveloped, resulting in insufficient clamping force and failed grasping attempts. We used a single sheet of paper, a large acrylic plate, a bulky packing box, and a PCB (Printed Circuit Board) to test the gripper’s performance. MST adhesion provided a strong gripping force for flat objects, while the active detachment mechanism enabled precise placement after grasping. The results confirm that MST-G exhibits remarkable adaptive grasping capabilities, successfully handling both flat objects and various irregularly shaped items found in everyday life.

### 6.3. Adaptive Perching Experiments

**Figure 11:**
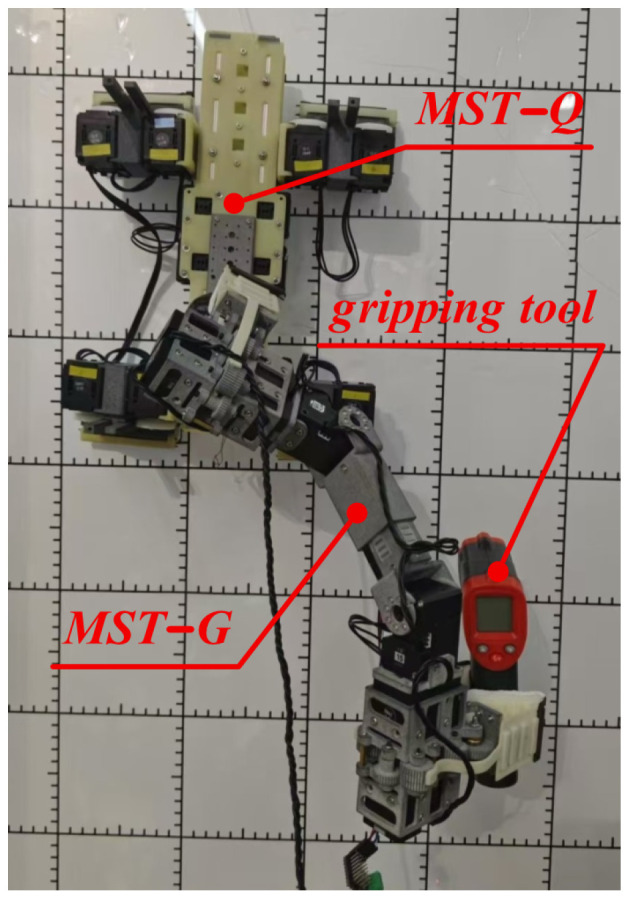
Adaptive perching and grasping experiments with MST-G.

To further validate MST-G’s adaptive perching capability, the MST-Q [30] dry adhesive quadruped climbing robot was used as the perching body, and the MST-G was perched to the back of the dry-adhesive quadruped climbing robot, and the temperature gun is grasped and moved to the desired position. These experiments prove that MST-G robot is capable of adaptive perching and grasping. The specific perching experiments are shown in Figure 11.

### 6.4. Climbing Experiments

**Figure 12:**
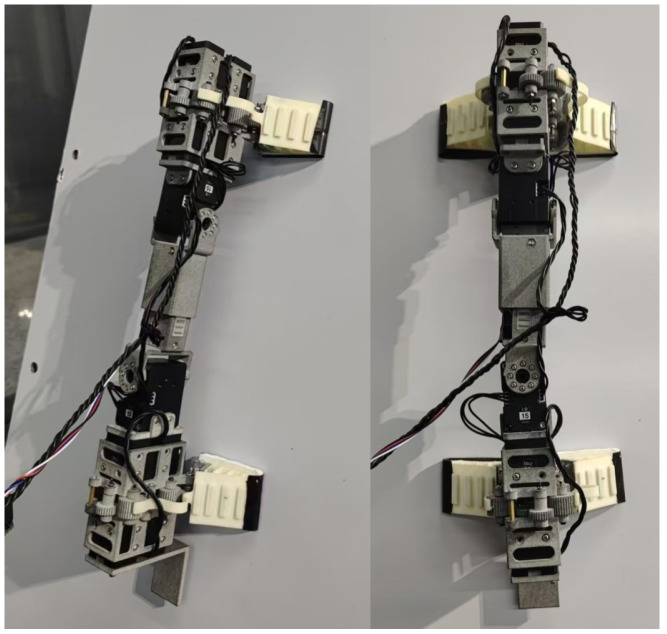
MST-G stabilized climbing on a 60° incline.

To evaluate the autonomous climbing capability of MST-G, we employed a smooth 60° inclined surface as the climbing platform for performance assessment, as shown in Figure 12. MST-G utilizes an active detachment gripper to achieve adhesion and detachment. The adhesion process secures the robot to the climbing surface, while the detachment process allows the gripper to detach from the surface. The linear actuator then drives MST-G upward through linear motion. By repeating these steps, MST-G achieves autonomous climbing movement. The experiments showed that MST-G can move stably on this surface.

## 7. Conclusions

To enhance the adaptive grasping capabilities of dry adhesion based climbing robots, this paper introduces a novel dry adhesion climbing robot, MST-G, which features autonomous climbing, perching, and flexible adaptive grasping. During operation, MST-G is integrated with a legged climbing robot to perform tasks, while in idle periods, it autonomously climbs, thereby reducing the robot’s load and ensuring stable operation. Additionally, a robust controller based on prescribed performance has been developed to ensure accurate position tracking, and its effectiveness has been validated through tests on MST-G. The robot is capable of adaptive perching on various objects and can perform grasping tasks on both irregularly shaped and flat objects, as well as autonomously climbing surfaces inclined at 60 degrees. In future research, efforts will focus on further optimizing the overall structure to enhance the adhesive performance and adaptive grasping capabilities of the active detachment gripper while maintaining its output capacity, aiming to achieve climbing on vertical and inverted surfaces.

## Figures and Tables

**Table 1:** DH Kinematic Parameters Table.

i	$\theta_{i}$ (rad)	$d_{i}$ (m)	$a_{i}$ (m)	$\alpha_{i}$ (rad)	Range (rad/m)
1	$\theta_{1}$	$l_{1}$	0	0	$\left(-\frac{\pi }{2},\frac{\pi }{2}\right)$
2	0	$l_{2}$	0	$-\frac{\pi }{2}$	(0,0.03)
3	$\theta_{3}$	0	0	0	$\left(-\frac{\pi }{2},\frac{\pi }{2}\right)$
4	0	$l_{4}$	0	$-a_{4}$	/

## Data Availability

Data are contained within the article.
